# Do Boys and Girls Evaluate Sexual Harassment Differently? The Role of Negative Emotions and Moral Disengagement

**DOI:** 10.3390/bs15101306

**Published:** 2025-09-24

**Authors:** Laura Bosaia, Gemma Garbi, Elisa Berlin, Camilla Lasagna, Loredana Macrì, Maria Noemi Paradiso, Norma De Piccoli

**Affiliations:** CIRSDe (Centro Interdisciplinare di Ricerca e Studi delle Donne e di Genere—Interdisciplinary Center for Research and Studies of Women and Gender), Dipartimento di Psicologia (Psychology Department), University of Turin, 10153 Torino, Italy; laura.bosaia@unito.it (L.B.); elisa.berlin@unito.it (E.B.); camilla.lasagna@unito.it (C.L.); loredana.macri@unito.it (L.M.); marianoemi.paradiso@unito.it (M.N.P.); norma.depiccoli@unito.it (N.D.P.)

**Keywords:** sexual harassment, adolescents, moral disengagement, emotional responses, gender differences

## Abstract

Adolescents’ perception and recognition of sexual harassment (SH) are shaped by several psychosocial variables, including gender norms, emotional responses, and ideological beliefs (such as sexism). This study specifically aimed to investigate the mediating roles of moral disengagement and emotional responses in the relationship between tolerance of SH and recognition of harassment scenarios, while considering gender as a moderator. The sample included 380 high-school students (55.3% female, 44.7% male), aged between 14 and 18 years (M_age_ = 15.71, SD_age_ = 0.87). No significant direct association was found between attitudes toward sexually harassing behaviour (TSHI) and recognition of potential harassing scenario (assessed by the Sexual Harassment Definitions Questionnaire—SHDO). However, TSHI was indirectly associated with SHDO through two distinct mediational pathways. On the one hand, higher tolerance of sexual harassment was associated with increased moral disengagement, which in turn was related to lower recognition of SH. On the other hand, it was associated with reduced negative emotional reactions, which were in turn associated with greater recognition of harassment. Additionally, gender differences emerged: females demonstrated greater ability to identify harassment scenarios and reported stronger negative emotional reactions. Overall, these findings highlight the role of psychosocial mechanisms in shaping adolescents’ recognition of harassment situations.

## 1. Introduction

Sexual harassment in adolescents is a pervasive public health problem (Wood et al., 2025). Between 30% and 48% of adolescents experience sexual harassment victimization (Clear et al., 2014; Hill & Kearl, 2011) and up to 80% declare having suffered some form of victimization during their school career (Lipson, 2001). Basile et al. (2020) report, about physical dating violence and sexual dating violence, that “10.8 percent reported sexual violence by anyone, with 50 percent of cases by a perpetrator other than a dating partner; 19.5% reported bullying in school, and 15.7 percent reported electronic bullying victimization during the past year” (Sakellari et al., 2022, p. 2).

If we compare international studies we can observe a high heterogeneity (Sánchez-Jiménez & Muñoz-Fernández, 2021): a meta analytic review by Wincentak et al. (2017) reports rates between 1% and 61% for physical aggression and the review of Stonard et al. (2014) shows between 20% and 77% for psychological aggression.

This high heterogeneity depends on the different forms of sexual harassment considered in the studies, the age of the subjects, cultural differences, and the type of relations (at school; romantic; with peer, etc.).

Sexual harassment (SH) is a multifaceted form of gender-based violence that affects individuals across all ages and contexts. Traditionally studied within workplaces and adult environments (Fitzgerald et al., 1995; Karami et al., 2021), SH is increasingly recognized as a critical issue in adolescence, where it is often underreported and misrecognized. According to the systematic literature review by Karami and colleagues (2021), youth bullying and victimization and perceptions of sexual harassment have emerged as two of the most rapidly growing areas of research in recent years, reflecting a shift toward understanding SH in adolescent and educational settings.

Adolescents are particularly vulnerable to SH, as this developmental phase involves the internalization of gender norms, identity construction, and the emergence of romantic and sexual experiences. Several studies suggest that sexually harassing behaviours frequently occur among peers in school settings (Hill & Kearl, 2011; McMaster et al., 2002; Vega-Gea et al., 2016), but are often misinterpreted or normalized through peer culture and social scripts (Foulis & McCabe, 1997; Fernández-Fuertes et al., 2020).

Despite a growing interest, a significant gap remains in understanding how adolescents perceive and interpret experiences of sexual harassment, particularly outside adult-centric legal and occupational frameworks (Sakellari et al., 2022). Moreover, existing studies tend to focus primarily on victimization within romantic or dating relationships (Fernández-Fuertes et al., 2020) and only a small number of studies aim to identify the constructs that underlie the justification of harassment, regardless of whether or not there is experience of victimization. Bolduc and colleagues (2022), in their scoping review, categorize the correlates of adolescent sexual harassment victimization into individual-level factors—such as trauma history and attitudes—and contextual-level factors—such as peer norms and school climate—underscoring the importance of multifactorial explanatory models.

Recent studies have also emphasized how victimization and tolerance of SH are shaped by affective and cultural scripts in adolescents’ dating experiences. Drawing on the ambivalent sexism, Ayala and colleagues (2021), in the Lights4Violence European baseline, found that both hostile and benevolent sexism are prevalent among 12–17-year-olds—boys displaying higher levels than girls—and that higher maternal education correlates with lower benevolent sexism among girls. Cuadrado-Gordillo and Martín-Mora-Parra (2022) highlight that in more traditional cultural contexts, hostile sexism is higher, and that benevolent sexism influences the perceived severity of violent behaviours, acting as a mediator between victimization and the normalization of abuses. Arrojo and colleagues (2024) validated the Perceived Severity of Adolescent Dating Violence (PS-ADV) scale, showing that higher perceived severity of dating violence is associated with lower victim-blaming attitudes, lower ambivalent sexism, and greater empathy—factors that may enhance the recognition of SH in romantic contexts.

In synthesis, many studies demonstrate links between victimization, tolerance, and perpetuation of SH in adolescent romantic and dating relationships. Fewer studies have been devoted to identify the psychological and cultural factors that influence sexual harassment attitudes, since attitudes can predispose individuals to action. Clarifying the psychosocial processes underlying sexual harassment attitudes could inform the development of targeted training programmes, which are one way to prevent such behaviour.

### 1.1. Sexual Harassment Perception Among Adolescents

Perception and recognition of SH in adolescence are shaped by multiple psychosocial factors, including gender norms, emotional responses, and ideological beliefs. A growing body of research shows that adolescents do not always interpret potentially harassing situations as inappropriate or harmful. As highlighted by Sakellari and colleagues (2022), adolescent definitions of SH vary considerably across gender and cultural contexts: while some behaviours are clearly identified as unacceptable by most respondents (e.g., physical aggression), others, such as sexual jokes or unwanted compliments, are interpreted ambiguously, especially when perpetrated by peers or framed as jokes. Similarly, Sweeting and colleagues (2022), in a mixed-methods study conducted in Scottish secondary schools, found that students struggled to distinguish between what they called “banter” and what could be described as SH, depending on intent, context, and perceived harm.

These ambivalences are not merely semantic, but reflect deeper psychological and normative mechanisms. A benevolent form of sexism is embedded in our culture (Glick & Fiske, 1996). Like hostile sexism, it serves to justify women’s subordinate status, albeit in a more protective and seemingly positive manner. It is more difficult to identify because it is expressed through kind behaviours and favourable evaluations that nonetheless reinforce gender stereotypes. In contrast, hostile sexism constitutes an overt expression of discrimination that relegates women to a subordinate role. In the context of adolescent relationships, sexism has been associated with cognitive, emotional, attitudinal and behavioural aspects (see the systematic review of Ramiro-Sánchez et al., 2018). Due to its ambivalent nature, the distinct functions of hostile and benevolent sexism must be clearly delineated (see Russell & Trigg, 2004; Ayala et al., 2021; Cuadrado-Gordillo & Martín-Mora-Parra, 2022). In adolescence, hostile sexism has been empirically shown to increase following romantic relationship experiences—particularly among girls (De Lemus et al., 2010)—suggesting a potential pathway through which such attitudes may support the tolerance of sexually harassing behaviours.

Leaper and Gutierrez (2024) emphasize how sexism—particularly in its hostile and prescriptive forms—continues to shape adolescent attitudes toward gender-based abuse, fostering tolerance for aggressive male behaviour and victim blaming attitudes toward female targets. These gendered expectations may affect how behaviours are interpreted: for example, boys may be less likely to label certain acts as harassment, while girls may be more likely to blame themselves or downplay their discomfort in order to maintain social harmony (Vega-Gea et al., 2016).

Among the most salient psychosocial dimensions associated with adolescents’ recognition of SH, moral disengagement stands out as a particularly relevant variable, as it helps elucidate the various forms that the harassment justification can take. Bandura’s (1999) theory of moral disengagement explains how individuals justify or minimize morally reprehensible behaviours, such as SH, by altering their interpretation through denial, displacement of responsibility, or dehumanization. This process allows adolescents to tolerate harassment without perceiving it as wrong.

Expanding on Bandura’s model, Page and Pina (2018) developed a domain-specific measure of moral disengagement in SH, the Moral Disengagement in Sexual Harassment Scale, which identifies mechanisms such as euphemistic labelling, denial of injury, and victim blaming. Adolescents who endorse such justifications are less likely to recognize SH scenarios or consider them morally wrong. These processes interact with broader ideological constructs, such as hostile sexism, which legitimizes SH through narratives of provocation, naturalization of male behaviour, or male entitlement to female attention.

In addition to cognitive and ideological dimensions, emotional responses play a crucial role. Emotional reactions, such as feeling flattered versus annoyed or scared, significantly influence whether adolescents label a behaviour as sexual harassment—a dynamic supported by empirical studies showing that negative emotions often accompany perceived harassment, while ambiguous or non-harassing situations may elicit neutral or positive responses (Peled-Laskov et al., 2020). Fernández-Fuertes and colleagues (2020) found that emotional ambivalence and romantic myths (e.g., interpreting jealousy as a sign of love) can lead adolescents to reinterpret abusive dynamics as normal or even desirable in the context of dating relationships.

Taken together, these findings indicate that the ability to recognize SH is not merely a matter of information or awareness, but is embedded in a broader system of values, emotions, and social expectations. The present study aims to investigate how these elements interact to shape adolescents’ recognition of SH scenarios. Particular attention is given to the Italian context, where the normalization and trivialization of sexual harassment among youth remain underexplored. While sexual harassment among adolescents has been studied extensively in Anglo-Saxon contexts, there is a lack of empirical research exploring how these dynamics are perceived and normalized in the Italian socio-cultural landscape, especially in relation to digital spaces and affective scripts.

### 1.2. The Italian Context: Some Data

Because recent comparative data on adolescent sexual harassment are lacking, we provide a brief overview of Italy’s gender equality performance relative to other European countries, which we might consider an indirect expression of persistent stereotypes and a consequence of forms of sexism.

Italy ranks 14th in the EU on the Gender Equality Index, out of 28 countries, with a score of 69.2 points out of 100 (1.8 points below the EU score). Regarding violence against women, 41% of Italian women and 35% of European women aged 18–29 report having experienced physical and/or sexual violence by any perpetrator since the age of 15 (EIGE, 2024). These data do not specify the prevalence of sexual harassment among adolescents, but they do offer a snapshot of how European countries address broader gender-related issues.

Italy is a country with opposing views: on the one hand, innovative lifestyles rooted in openness, acceptance, and progress; on the other, civil and political perspectives that reinforce traditional values and conformity. These contrasting positions are also observed in debates over sexual and emotional education. In particular, in the Italian context, where public discourse on sexual violence has intensified following movements such as #QuellaVoltaChe (“That Time”) and #MeToo, there is a growing need to explore how these dynamics are experienced during adolescence. This developmental stage is characterized by a progressive process of separation from the family unit, during which adolescents form affective and romantic bonds that are central to the construction of their identity. The ways in which these relationships are built—and the implicit or explicit norms that regulate them—strongly shape how adolescents perceive consent, boundaries, and violence.

Recent national data provide meaningful insights into how Italian adolescents interpret sexual violence and harassment. The report Le Ragazze Stanno Bene? (“Are the Girls OK?”), published in 2024 by Save the Children in collaboration with IPSOS (Save the Children & IPSOS, 2024), presents findings from a representative survey of 800 adolescents aged 14 to 18, stratified by gender, age, and geographic area. The study paints a picture of adolescence in which gender stereotypes remain deeply rooted: for example, 70% of respondents believe that girls are more prone to crying, and 50% think that females are better suited to caregiving roles. Only 26% believe that boys and girls are equally likely to cry. When it comes to romantic relationships, 30% of adolescents consider jealousy a form of love, and around 20% believe that certain controlling behaviours within a relationship can also be expressions of love. Moreover, 43% of adolescents—regardless of gender—believe that if a girl does not want to have sex with someone, she will be able to find a way to avoid it, reflecting a problematic view of sexual consent.

A significant proportion of adolescents report having either experienced or perpetrated gender-based violence within intimate relationships: 41% have suffered violent behaviours, while 30% admit to having engaged in them. The most commonly reported forms of perpetrated violence include: repeatedly calling a partner to find out where they are (29%), using violent language such as shouting or insults (27%), and engaging in physically intimidating behaviours (such as slapping, punching, or pushing) (15%).

These are some data that indicate that many adolescents—particularly girls—report having witnessed or experienced behaviours that could be categorized as sexual harassment, yet often struggle to identify these acts as such. This difficulty in labelling and interpreting violence is echoed in the Senza Confine—Survey Teen 2024, conducted by Fondazione Libellula (2024), which highlights adolescents’ ambivalence and uncertainty in recognizing harassment, especially within peer and romantic contexts.

The Violenza Onlife report, published in 2024 by Save the Children (2024) and involving an adolescent sample, further expands this picture by highlighting how violence—including harassment and coercion—increasingly takes place in digital spaces. Specifically, a substantial proportion of participants reported having received unsolicited sexual messages, images, or pressure to send intimate content online, particularly through messaging platforms and social media^1^. These experiences often go unreported and are not always perceived as forms of violence, confirming a broader difficulty in naming and recognizing digital sexual harm.

Taken together, these reports underscore the urgency of addressing how sexual harassment is recognized, interpreted, and often normalized in adolescence. These findings suggest the urgency of designing comprehensive educational interventions that target adolescents’ moral reasoning, emotional literacy, and critical awareness—both in offline and digital spaces—to prevent the normalization of sexual harassment.

In 20 European countries, sex education is mandatory in schools, although curricula and approaches differ. Italy is among the countries without mandatory sex education (along with Bulgaria, Cyprus, Lithuania, Poland, and Romania) (OnuItalia, 2023). In Italy, sex and relationship education is left to the discretion of individual schools and teachers, as no national decree mandates or promotes its inclusion across all educational levels. To date, a draft law on sex education in schools requires explicit parental consent, a point harshly criticized by minority parties, associations, and a segment of civil society. Meanwhile, alternative proposals have been presented to the Italian government, but none have yet been implemented. Nevertheless, a significant segment of public opinion supports the introduction of these topics in schools. A recent survey, published online (Coop, 2025), involving approximately 2000 people, found that 70% would like mandatory emotional education in schools, and 9 out of 10 Italians believe that school-based teaching can help reduce hatred, marginalization, and even gender-based violence.

Although a part of civil society desires that children, adolescents, and young people receive education on these issues, political and ideological obstacles and resistance remain strong in Italy.

### 1.3. The Present Study

The aim of the present research was to investigate how adolescents recognize situations of sexual harassment, and how this recognition was influenced by sexist beliefs (e.g., attitudes towards SH, Benevolent and Hostile Sexism), moral disengagement, and emotional responses. This study adopts an integrated psychosocial framework, drawing on theories of moral disengagement (Page & Pina, 2018), ambivalent sexism (Glick & Fiske, 1996), and emotional responses in adolescent interpretations of sexual harassment (Sweeting et al., 2022). In this study, sexual harassment recognition is operationalised as participants’ ability to identify behaviours as harassment, referred to 10 proposed scenarios, following the Sexual Harassment Disclosure and Recognition Questionnaire (SHDO; Foulis & McCabe, 1997). While emotional reactions (e.g., anger, fear, sadness) are operationalised as participants’ self-reported affective responses for each of the proposed scenarios (Foulis & McCabe, 1997).

**Figure 1:**
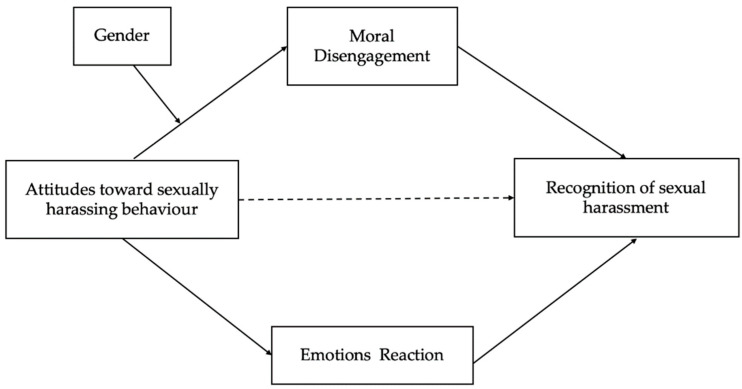
Hypothesized model.

While previous research has mainly focused on adults, there is a growing need to explore these dynamics among adolescents, particularly in light of the rising public and educational concern around gender-based violence in the Italian context. However, existing studies have typically examined these processes in isolation, considering either cognitive mechanisms such as moral disengagement or emotional responses, but not both simultaneously. To our knowledge, this is the first study to test the dual mediating role of emotions and moral disengagement in adolescents’ recognition of sexual harassment, while also including gender as a moderating factor. By integrating these dimensions, we extend existing theoretical frameworks and offer a novel contribution to understanding how adolescents interpret and respond to harassment scenarios. Specifically, we aimed to assess the role of moral disengagement and emotional responses as two mediators of the relationship between tolerance towards SH and recognition of harassment scenarios (Figure 1). Furthermore, since all studies have demonstrated gender differences, with girls expressing less tolerance for sexual harassment (Foulis & McCabe, 1997; Bolduc et al., 2022), less moral disengagement (Sánchez-Jiménez & Muñoz-Fernández, 2021), and less sexism (Sánchez-Jiménez & Muñoz-Fernández, 2021) than their male peers, we intend to examine whether tolerance and recognition of sexual violence differ between boys and girls. In particular, we expected that:

**H1.**  *Higher tolerance towards SH would be associated with increased levels of moral disengagement, which in turn would reduce adolescents’ ability to label ambiguous scenarios as harassment.*

**H2.**  *Higher tolerance towards SH would be associated with weaker negative emotional responses, which could undermine the intuitive moral appraisal of the situation and thus hinder accurate recognition.*

In addition, drawing on the literature highlighting the subtle yet pervasive influence of benevolent sexism (e.g., Russell & Trigg, 2004; Sánchez-Jiménez & Muñoz-Fernández, 2021; Cuadrado-Gordillo & Martín-Mora-Parra, 2022), we hypothesized a direct negative association between benevolent sexism and harassment recognition (H3). However, this hypothesis was not tested due to the low reliability of the benevolent sexism scale (for details, see Section 2.2). We expected the following:

**H3.**  *Higher levels of benevolent sexism would be negatively associated with adolescents’ ability to recognise harassment scenarios*.

Finally, based on the aforementioned research literature suggesting gender differences in the perception and interpretation of gender-based violence, we explored gender as a potential moderator. In particular, we expected that:

**H4.**  *Gender will moderate the indirect effects of tolerance toward sexual harassment on recognition.*

## 2. Materials and Methods

### 2.1. Participants and Procedure

Data were collected between December 2024 and February 2025 and consisted of an anonymous online questionnaire, which took approximately 35 min to complete. The students were recruited from public secondary schools in the North of Italy. To identify the schools, CIRSDe (University of Turin) sent a communication, through the Regional School Office to all secondary schools in the city to describe an education project focused on relationships, which included a questionnaire to be administered before the intervention. At the same time, schools were invited to express their interest in implementing the project. The four schools that expressed interest in realizing the project, subsequently selected eight classes each for the questionnaire administration (16 classes participated in the project—experimental group—and 16 classes only completed the questionnaire—control group). Before completing the questionnaire, the schools involved obtained informed consent, signed by both parents (for students under 18) and the students themselves. The questionnaire was not administered to those who did not provide consent.

Before answering the questionnaire, participants read an informed consent and were informed about their right to refuse to participate in the study and to discontinue the study at any moment without any consequences. All the students were also informed that their anonymity would be guaranteed. No compensation was given for their enrolment. The study followed the ethical principles of the 1964 Declaration of Helsinki. The study procedures were approved by the Bioethics Committee of University of Turin (protocol n. 0621943, 30 October 2024).

Of the 470 students, 80.85% completed the questionnaire in each section. The final sample consisted of 380 high-school students (55.3% female, 44.7% male), aged between 14 and 18 years (M_age_ = 15.71, SD_age_ = 0.87; 7 cases were missing for this variable). Most of them (90.1%) were born in Italy and have not failed any school year (88.2%).

### 2.2. Measures

All measures were translated from English into Italian using the back-translation procedure. The following measures were included in the questionnaire:

- The Ambivalent Sexism Inventory for Adolescent—Short Version (ISA; De Lemus et al., 2010). A subset of six items was selected based on item wording and their theoretical coverage of the three dimensions of Hostile Sexism. In particular, three items measured Hostile Sexism toward women (e.g., “Boys should exert control over who their girlfriends interact with”; α = 0.70) and 3 items measured Benevolent Sexism toward women (e.g., “Girls should be cherished and protected by boys”). Due to the low reliability, the Benevolent Sexism subscale was not used in the following analysis. The items were rated on a 6-point Likert-type scale ranging from “strongly disagree” (0) to “strongly agree” (5).
- The eight-item version of the Moral Disengagement in Sexual Harassment Scale (MDiSH; Page & Pina, 2018). Each item, one per moral disengagement mechanism, was adapted to the school context (e.g., “In a study place with a relaxed atmosphere, men cannot be blamed for “trying it on” with attractive girls when they get the chance”; α = 0.80). These items were rated on a 7-point Likert-type scale (1 = strongly disagree, 7 = strongly agree).
- The Sexual Harassment Definitions Questionnaire (SHDO; Foulis & McCabe, 1997). It consists of 10 scenarios of potential sexual harassment, including 5 same-gender harassers and 5 opposite-gender scenarios. For each scenario, participants were asked to indicate whether they considered it to be a case of sexual harassment (yes = 1; no = 0). Higher scores (range: 0–10) indicate a greater ability to recognize and label cases as sexual harassment.
- The Emotional Reactions to Harassment Scenarios (from Foulis & McCabe, 1997). For each of the 10 scenarios included in the SHDO (Foulis & McCabe, 1997), participants were also asked: “If you were [name of the protagonist], would you feel: flattered, annoyed, worried, appreciated, not bothered?”. Participants could select each of these five emotional reactions per scenario. Responses were coded dichotomously (1 = emotion selected; 0 = not selected). Although this emotional reaction question was included in the original SHDO questionnaire, the original article did not report any analyses of these responses.
- The Attitudes Toward Sexually Harassing Behavior (TSHI; Lott et al., 1982; α = 0.73) was used to assess respondents’ general attitudes toward SH. It contains 10 items (e.g., “It is only natural for a man to make sexual advances to a woman he finds attractive”) and measures on a 5-point Likert-type scale (1 = strongly disagree, 5 = strongly agree). Two items were reversed so low values indicate high tolerance for SH.

Finally, a brief list of socio-demographic items was administered (i.e., sex and year of birth). Additional measures were administered for exploratory purposes but are not considered in the present research. These variables are beyond the scope of the present study and will be analysed in future work.

### 2.3. Data Analyses

Analyses were conducted using IBM SPSS Statistics version 29. Descriptive statistics (i.e., mean and standard deviation), independent sample *T*-test, and bivariate correlations between the key variables were performed. Cohen’s D was used to assess the effect size for the significant differences between boys and girls, using the following criteria: small effect size (d < 0.20); medium effect size (d > 0.50); large effect size (d > 0.80); very large effect size (d > 1.30) (Cohen, 2013).

**Figure 2:**
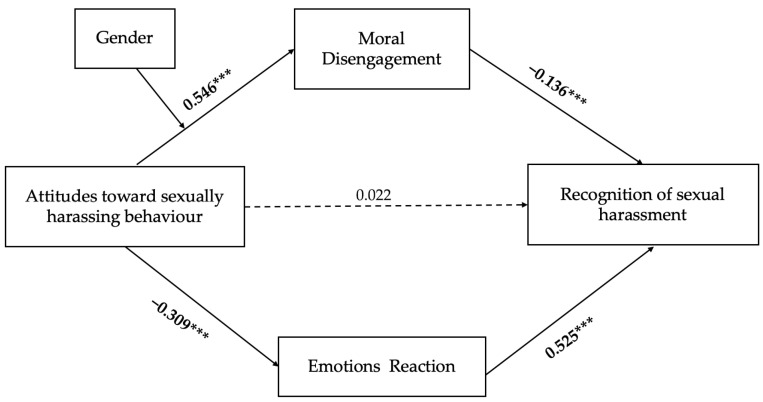
Mediational model. Note: Standardized coefficients are presented. Bolded coefficients are statistically significant. *** *p* < 0.001.

A cumulative score was calculated for each emotion. Then, based on theoretical and empirical considerations, these responses were categorized as either negative (annoyed, worried) or positive emotions (flattered, appreciated). This pattern was further confirmed by a post hoc exploratory factor analysis (EFA), which revealed a clear two-factor structure corresponding to negative and positive emotional responses. For each participant, a cumulative score of negative/positive emotional responses was calculated. Higher scores indicate a stronger tendency to experience negative/positive emotional reactions when imagining oneself in the position of the victim. An exploratory descriptive analysis revealed that positive emotions were rarely selected by participants and showed consistently low mean scores. Based on these results, Positive Emotional Responses were excluded from further analysis. Consequently, the model test focused on negative emotional reactions and moral disengagement, as shown in Figure 2. The mediation model, as well as the moderation mediation, was performed using Hayes’ PROCESS macro (Version 4.1, Model 4 and 7). The indirect effects hypothesized were assessed using bootstrap estimation (Hayes, 2019) with 5000 samples. The bias-correct 95% confidence interval (CI) was calculated by determining the effects at the 2.5th and 97.5th percentiles. The indirect effects were statistically significant when 0 was not included in the CI.

## 3. Results

### 3.1. T-Tests and Correlations

Table 1 shows the descriptive statistics and the bivariate correlations of the variables. All study variables were significantly correlated with each other. Specifically, Hostile Sexism was positively associated with Attitudes toward sexually harassing, Moral Disengagement and Positive Emotion Reactions. In contrast, Negative Emotion Reactions were negatively correlated with all the variables except Recognition of sexual harassment (SHDO). Additionally, TSHI was positively associated with Moral Disengagement and Positive Emotion Reaction, while negatively related to Recognition of sexual harassment (SHDO). This variable was also negatively associated with Hostile Sexism (HS), Moral Disengagement and Positive Emotion. Finally, increases in levels of Moral Disengagement were correlated with increases in Positive Emotion Reactions.

As shown in Table 1, sexism expressed by participants was quite low. Decidedly low was positive emotion reactions, while the ability to recognize harassment fell below the midpoint of the scale.

*T*-tests were performed to explore gender differences across all variables. As shown in Table 2, significant differences emerged for all variables considered. Except for TSHI and SHDO, which showed a medium effect size and a very large effect size respectively, the other group differences were large. Specifically, male participants scored higher than women on HS, Moral Disengagement in Sexual Harassment, Attitudes toward sexually harassing behaviour and Positive Emotion Reactions. Regarding SHDO, women showed a greater ability to recognize and label cases as sexual harassment and more Negative Emotion Reactions to Harassment Scenarios.

### 3.2. Mediation Model

To test our hypotheses, a mediation model was tested, using Model 4 of PROCESS macro. TSHI was entered as the independent variable, Recognition of sexual harassment as the dependent variable, and Moral Disengagement and Negative Emotion as mediators. Both H1 and H2 were supported.

As shown in Table 3, there was no significant direct association between Attitudes toward sexually harassing and Recognition (b = 0.022; SE = 0.20; *p* = 0.67). However, higher levels of TSHI predicted increased levels of Moral Disengagement (b = 0.55; SE = 0.11; *p* < 0.001) and lower negative emotional responses (b = −0.31; SE = 0.16; *p* < 0.001).

Importantly, the indirect effects were significant: increased levels of TSHI indirectly predicted a decreased level of SHDO through both Moral Disengagement (b = −0.07; Bootstrap SE = 0.11; 95% CI [−0.13, −0.02]) and Negative Emotion (b = −0.16; Bootstrap SE = 0.03; 95% CI [−0.23, −0.10]).

### 3.3. Moderated Mediation Model

To examine the role of gender as a moderator, a moderated mediation (Model 7 of PROCESS) was tested. As before, recognition of sexual harassment, was entered as the dependent variable. Attitudes toward sexually harassing was the independent variable, while Moral Disengagement was the mediator and Gender (1 = male, 2 = female) was the moderator. Supporting H4, the index of the moderated mediation was significant (Index = 0.40, SE = 0.16; 95% CI = [0.12, 0.73]), so the conditional effect of Attitudes toward sexually harassing on recognition via Moral Disengagement is moderated by gender. Specifically, as shown in Table 4, the indirect effect was significant and negative among male participants (B = −0.76; 95% CI [−1.11, −0.43]), and weaker but still significant among female participants (B = −0.36; 95% CI [−0.55, −0.20]). These results suggest that higher SHAS is associated with lower ability to correctly identify and label cases as sexual harassment through increased moral disengagement, especially among male adolescents.

## 4. Discussion

The present study examined how adolescents recognize scenarios of sexual harassment, and how such recognition is associated with attitudes towards harassment, moral disengagement, and emotional responses. Preliminary analyses revealed gender differences consistent with the existing literature on gender-based violence. Specifically, girls reported significantly lower levels of hostile sexism, moral disengagement, and tolerance towards sexual harassment compared to boys. Conversely, they demonstrated a greater ability to identify harassment scenarios and reported stronger negative emotional reactions.

Despite overall low mean scores on the positive emotions scale, boys reported significantly higher levels of positive emotional responses than girls. This finding may reflect gender norms that tend to minimize the seriousness of harassment, or even reinterpret it as something benign or flattering. Overall, these results align with prior research on gender-based violence, which highlights a greater female sensitivity to situations involving harassment; this is shown both among adults (Swim & Cohen, 1997; DelGreco et al., 2021) and adolescents (Vega-Gea et al., 2016; Ayala et al., 2021; Sakellari et al., 2022; Arrojo et al., 2024).

Due to low reliability, the benevolent sexism scale was not included in the mediation analyses. However, the hostile sexism component showed a positive correlation with more TSHI, higher levels of moral disengagement, and stronger positive emotional responses to the ambiguous scenarios. In contrast, it was negatively associated with both negative emotional reactions and SHDO. Importantly, the recognition of SH was positively correlated only with negative emotional responses and was negatively correlated with all other variables. This seems to suggest that emotional and cognitive factors may contribute in distinct ways to adolescents’ understanding of gender-based violence.

Contrary to our expectations, no significant direct association was found between TSHI and SHDO. However, as hypothesized, TSHI was indirectly associated with SHDO through two distinct psychological pathways. On the one hand, higher tolerance of sexual harassment (i.e., a pattern of beliefs that tends to downplay the seriousness of the phenomenon by portraying it as exaggerated or not truly problematic, or that normalises sexual advances towards girls as something to be expected or inevitable; Lott et al., 1982) was linked to increased moral disengagement, which in turn was related to lower recognition of SH. On the other hand, it was associated with reduced negative emotional reactions, which instead predicted better recognition. Regarding gender differences, the results confirmed that the indirect effect of attitudes towards harassment on recognition, mediated by moral disengagement, was significantly moderated by gender. Specifically, among boys, more tolerant attitudes were associated with higher levels of moral disengagement, which in turn were linked to a reduced ability to recognize harassment scenarios. Although this indirect effect was also observed among girls, it was significantly weaker.

This result can be better understood through the lens of gender socialization theories (Fagot et al., 2012). From early adolescence, boys are often exposed to cultural scripts that emphasise competitiveness, dominance, and peer approval, which may normalise aggressive behaviours and minimize their consequences (Leaper & Gutierrez, 2024). In this sense, moral disengagement may function as a mechanism through which boys reconcile socially transmitted ideals of masculinity with behaviours that would otherwise be morally questionable. This interpretation is consistent with meta-analytic evidence showing that boys are more likely to adopt justificatory mechanisms to legitimise aggression (Gini et al., 2014). By contrast, girls’ comparatively weaker reliance on moral disengagement may reflect both greater sensitivity to interpersonal harm and gendered expectations of empathy and relational responsibility. Taken together, these dynamics highlight how moral disengagement interacts with gendered social norms, reinforcing the tendency among boys to reinterpret sexual harassment in less problematic terms. This gender-related pattern aligns with previous research showing that boys are more likely to morally disengage in peer or relational aggression contexts and are more susceptible to adopting justificatory mechanisms that normalize or minimize violent acts (Gini et al., 2014; Obermann, 2011).

In our model, moral disengagement emerged as a key mediator, suggesting that cognitive rationalizations may play a central role in weakening adolescents’ ability to recognize SH. While our cross-sectional design does not allow for causal claims, this interpretation aligns with previous research showing that the link between gender beliefs and aggressive behaviour can be shaped by normative beliefs and social approval processes (e.g., Reyes et al., 2016; Sánchez-Jiménez & Muñoz-Fernández, 2021). Future studies are needed to better understand how these mechanisms evolve over time and under which social conditions tolerant attitudes and disengagement may facilitate behavioural expressions of harassment or complicity.

Drawing on Moral Disengagement Theory (Bandura, 1999), our findings seem to suggest that adolescents who report more tolerant attitudes towards sexual harassment, especially males, tend to internalise relational scripts grounded in power asymmetries and male dominance, especially in interpersonal contexts. The observed higher propensity for moral disengagement among boys could be explained by the internalization of gender roles that valorise dominance and downplay the emotional impacts of aggression. This perspective is in line with recent studies focusing on the role of moral disengagement in dating aggression (i.e., a specific type of intimate partner violence that occurs in adolescent romantic relationships; it does not just involve physical violence but also sexual, psychological, and digital forms of aggression). For example, Sánchez-Jiménez and Muñoz-Fernández (2021) found that adolescents classified as highly morally disengaged and endorsing strong sexist attitudes exhibited significantly higher levels of both psychological and physical aggression towards their partners. These findings echo other research indicating that hostile sexism is frequently directly linked to aggressive behaviours in dating contexts, while benevolent sexism may serve a more complex role, potentially increasing vulnerability to victimization but sometimes acting as a protective factor against perpetrating aggression. In light of its multifaceted nature, the role of benevolent sexism requires further investigation.

Taken together, the results of this study highlight the importance of adopting a multidimensional approach to understanding the psychological mechanisms associated with adolescents’ recognition of sexual harassment. Specifically, it appears that the impact of tolerant attitudes towards harassment operates through two distinct but complementary psychological pathways: on the one hand, a cognitive processing that rationalizes and justifies harassment, reflected in the activation of moral disengagement mechanisms; on the other hand, a reduced negative affective response, which may limit the ability to fully grasp the seriousness and problematic nature of the situation. This dual pathway emphasises the need to go beyond purely cognitive factors and to also consider emotional dimensions when attempting to understand and counteract the normalization of gender-based violence among peers. This is particularly relevant given that adolescence represents a critical period for the formation of attitudes and moral schemas. In light of this, prevention interventions should adopt gender-sensitive strategies that not only aim to challenge tolerant attitudes but also actively engage boys. Educational programmes addressing these issues on both cognitive and emotional levels may prove particularly effective.

Finally, some limitations must be acknowledged. First, the cross-sectional nature of the data precludes causal inferences. Future longitudinal or experimental research could clarify the temporal ordering of these variables. Second, the sample was not randomly selected and participants were only from northern Italian regions, thus limiting the generalizability of the results. Moreover, while our measure of emotional responses was based on validated scenarios and the dichotomous coding followed the original procedure of the SHDO (Foulis & McCabe, 1997), this approach may have obscured more nuanced or ambivalent emotional processes. Future studies should consider alternative emotion measures, such as Likert-type scales, and provide a more fine-grained understanding of adolescents’ emotional experience. A further limitation concerns the exclusive reliance on self-report measures. Since sexual harassment is a sensitive topic, participants’ responses may have been influenced by social desirability bias (i.e., the tendency to answer in ways that conform to socially accepted norms rather than expressing one’s genuine views). This may have led participants to underreport tolerant attitudes or overreport negative emotional reactions, which could partially explain the relatively low mean levels of sexism and tolerance of SH observed in our sample.

Future studies could benefit from adopting multi-method approaches, such as or experimental vignette designs, to reduce the impact of desirability biases and obtain a more nuanced picture of adolescents’ attitudes and perceptions.

Moreover, cross-cultural studies would allow to specify the role that norms and cultural values play in the educational path of new generations, consolidating, or modifying, gender stereotypes which, in turn, contribute to justifying or underestimating sexual harassment.

Despite these limitations, the results offer some suggestions for the development of future educational interventions. We propose that sexual harassment trainings programmes should also target moral disengagement and emotions underlying tolerance of SH by working to dispel myths about rape, sexual harassment, and challenge sexist beliefs. Future longitudinal studies will be essential to assess whether such interventions have sustained effects over time.

## 5. Conclusions

It is well established that attitudes and beliefs play a crucial role at the base of the Pyramid of Violence. These psychosocial dimensions have been widely studied, especially in adult populations (for updated data on gender stereotypes, see Eurobarometer, 2024, and FRA, 2024, on gender-based violence).

International data reveal widespread behaviours that reflect gender stereotypes, often contributing to the justification—or even the normalization—of violence, aggression, and harassment. However, when it comes to adolescents and young people, we lack comparable studies. Existing reports tend to focus on bullying, cyberbullying, and violence against children and adolescents, but there is a scarcity of data on how young people perceive and interpret sexual harassment. As Tomaszewska and Schuster (2021) point out, when it comes to teen dating violence (TDV), “some efforts should be made in providing representative data on TDV in Europe” (p. 22).

To better understand the mechanisms that shape attitudes, beliefs, and behaviours, it is essential to design educational pathways. In our study, for example, we did not find a direct relationship between attitudes toward sexually harassing behaviour and the recognition of harassment itself. This suggests the importance of addressing not only moral disengagement, but also of fostering emotional awareness, connecting cognitive and emotional dimensions in educational work.

According to Venäläinen (2025), “existing research suggests that young people’s views on sexual harassment might indeed have shifted in recent years, but these shifts may inform women and girls’ understandings to a greater extent than men’s and boys’” (p. 3). As our data show, gender differences persist, and education must play a key role. The Italian context is particularly critical in this regard. It is therefore essential to propose educational pathways that encourage dialogue and mutual understanding between boys and girls. Girls should be given space to articulate their experiences, difficulties, and fears—such as walking alone at night or being subjected to catcalling—while boys should be encouraged to listen and reflect on these everyday realities experienced by their female peers.

Ultimately, the goal is not only to avoid harmful behaviour, but to foster active citizenship, where all young people—regardless of gender—become engaged actors in preventing violence, supporting those who experience harm, and avoiding secondary victimization.

As reported by Euronews (2022), “Italy is one of the last member states in the European Union where sex education is not mandatory in schools. The main problem is ignorance, in the etymological sense of the word.” Indeed, Italy still lacks a national law mandating emotional and sexual education in schools. A proposal for such a law is currently under discussion but has faced delays, revisions, and repeated setbacks. As a result, the provision of emotional and sexual education is left to the discretion of individual schools.

Yet, emotional and sexual education must be seen as deeply interrelated—especially during adolescence, when young people begin to explore romantic relationships. These early experiences serve as a testing ground for developing relationship skills, navigating conflict, exploring seduction and rejection, and learning to build respectful relationships with oneself and others. For example, educational programmes could include training modules based on harassment scenarios (e.g., guided case analysis), as well as reflective activities on emotional reactions.

A shift in perspective is needed: instead of focusing solely on the responsibility of the perpetrator, or framing violence as a “private matter,” we must cultivate a sense of collective responsibility (Sulla et al., 2025), in which all citizens—including adolescents—are empowered as agents of cultural and relational change.

UNESCO and the World Health Organization promote sexuality education as a right to health, fundamental to gender equality and respect for human rights, which are among the UN’s sustainable development goals of the 2030 Agenda.

In conclusion, it is necessary to plan not only tertiary prevention interventions, but also programs that promote well-being, through educational pathways grounded in respect, empathy, and the acceptance of difference.

## Figures and Tables

**Table 1:** Descriptive statistics and bivariate correlations.

	M	SD	1	2	3	4	5
Hostile Sexism	1.29	1.09					
2. TSHI	2.24	0.65	0.45 **				
3. Moral Disengagement	2.44	1.11	0.62 **	0.55 **			
4. Negative Emotion Reactions	4.72	1.68	−0.36 **	−0.31 **	−0.42 **		
5. Positive Emotion Reactions	0.95	1.10	0.37 **	0.39 **	0.44 **	−0.29 **	
6. SHDO—Recognition of sexual harassment	4.55	2.48	−0.25 **	−0.21 **	−0.34 **	0.58 **	−0.31 **

** *p* < 0.01; TSHI = Attitudes toward sexually harassing behaviour; SHDO = Sexual Harassment Definitions Questionnaire.

**Table 2:** Differences between men and women: Mean scores, t, and *p* values.

	Mean Scores (SD)				
	Girls	Boys	*t*	*p*	*Cohen’s D*
Hostile Sexism	0.83 (0.80)	1.88 (1.13)	10.49	<0.001	0.96
Moral Disengagement	1.91 (0.76)	3.12 (1.12)	12.47	<0.001	0.93
TSHI	2.10 (0. 64)	2.50 (0.60)	6.18	<0.001	0.62
SHDO	5.10 (2.43)	3.90 (2.40)	−4.78	<0.001	2.42
Positive Emotion Reactions	0.61 (0.92)	1.36 (1.17)	7.08	<0.001	1.04
Negative Emotion Reactions	5.18 (1.60)	4.16 (1.62)	−6.13	<0.001	1.61

TSHI = Attitudes toward sexually harassing behaviour; SHDO = Sexual Harassment Definitions Questionnaire.

**Table 3:** Summary of direct, indirect, effects.

	β	B	SE	LLCI	ULCI
TSHI → SHDO	0.022	0.086	0.201	−0.309	0.480
TSHI → Negative Emotion	−0.309 ***	−0.800 ***	0.159	−1.113	−0.487
TSHI → MD	0.546 ***	0.929 ***	0.111	0.712	1.146
Negative Emotion → SHDO	0.525 ***	0.775 ***	0.065	0.648	0.902
MD → SHDO	−0.136 **	−0.306 **	0.123	0.013	−0.547
Indirect effects					
TSHI → MD → SHDO	−0.074	−0.284	0.111	−0.131	−0.015
TSHI → Negative Emotion → SHDO	−0.162	−0.620	0.032	−0.226	−0.103

TSHI = Attitudes toward sexually harassing behaviour; SHDO = Sexual Harassment Definitions Questionnaire; MD = Moral Disengagement; *** *p* < 0.001; ** *p* < 0.01; *p*-value not available for indirect effects.

**Table 4:** Moderated Mediation.

	Coefficient	SE	LLCI	ULCI
Outcome: Moral Disengagement				
TSHI	1.595 ***	0.271	1.061	2.129
Gender	0.351	0.372	−0.381	1.084
TSHI × Gender	−0.551 **	0.171	−0.888	−0.214
Outcome: SHDO		Conditional indirect effects		
	EFFECT	BootSE	BootLLCI	BootULCI
Male	−0.758	0.174	−1.108	−0.430
Female	−0.358	0.088	−0.546	−0.204

*** *p* < 0.001; ** *p* < 0.01; Index of moderated mediation = 0.400, Boot SE = 0.156, 95% CI [0.117, 0.729]; TSHI = Attitudes toward sexually harassing behaviour; SHDO = Sexual Harassment Definitions Questionnaire.

## Data Availability

For privacy reasons, the data presented in this study are available on request from the corresponding author.

## Note

1 Some data: the online exchange of intimate videos or photos occurs often in 14% of cases and rarely in 14%; sharing one’s intimate photos without consent occurs often in 6% of cases and rarely in 5% of cases. In essence, 54% of young people, with no difference between males and females, agree that “Those who send intimate photos always accept the risks they run, including that the photos may be shared with others”.
